# Determinants and Willingness to Pay for Purchasing Mask against COVID-19: A Protection Motivation Theory Perspective

**DOI:** 10.3390/ijerph19074268

**Published:** 2022-04-02

**Authors:** Qiying Ding, Shoufu Lin, Shanyong Wang

**Affiliations:** 1 School of Economics and Management, Dongguan University of Technology, Dongguan 523808, China; dingqy@dgut.edu.cn; 2 School of Economics, Fujian Normal University, Fuzhou 350007, China; 3 School of Public Affairs, University of Science and Technology of China, Hefei 230026, China; wsy1988@ustc.edu.cn

**Keywords:** COVID-19, mask, willingness to pay, protection motivation theory, contingent value method

## Abstract

Currently, coronavirus disease 2019 (COVID-19) is spreading globally, which poses great challenges to the whole world and human beings. The aim of this research is to understand the determinants and residents’ willingness to pay (WTP) for purchasing masks against COVID-19 in China. On the basis of protection motivation theory and contingent value method, this research shows that most residents are willing to purchase masks against COVID-19. COVID-19 knowledge, perceived severity, perceived vulnerability, and response efficacy are positively and significantly associated with residents’ WTP and the WTP value. However, self-efficacy is only significantly associated with residents’ WTP while not with WTP value. Furthermore, compared with other residents, residents in Hubei province have a higher level of COVID-19 knowledge, perceived severity, perceived vulnerability, self-efficacy and response efficacy, and the WTP value is higher. The average value of residents’ WTP value for purchasing masks against COVID-19 in Hubei province is ¥120.92 ($18.73) per month during the epidemic, while it is ¥100.16 ($15.50) for other residents. In addition, the effects of demographic factors such as age, gender, income, etc., on residents’ WTP and WTP value have also been examined.

## 1. Introduction 

At the end of 2019, a new coronavirus disease 2019 (COVID-19) suddenly broke out and pose unique challenges for everyone around the globe. Currently, COVID-19 has become a global public health event. On 11 March 2020, Dr. Tedros Adhanom Ghebreyesus, the Director General of the World Health Organization (WHO), announced that on the basis of scientific assessment, WHO believes that COVID-19 can be called a global pandemic. Almost all countries in the world have reported confirmed cases of COVID-19. By now, COVID-19 is still spreading around the world and showing no signs of improvement.

COVID-19 has caused a serious negative impact on the global economy and resident’s daily life. There is no doubt that the global economy will definitely decline. Furthermore, due to the infectivity of COVID-19 and its high fatality rate, COVID-19 also exerts a negative impact on resident’s physiological health, especially psychological health [1]. During the COVID-19 epidemic, residents more easily feel panic, anxious, scared, uneasy and worried that they can be infected [2]. To reduce its negative impact, several protective actions against COVID-19 can be taken by residents. For example, going out less and staying at home more, enhancing physical exercise and ensuring adequate sleep to enhance immunity, wearing masks, washing hands with medical alcohol, etc. It is noteworthy that these preventive measures can only prevent but cannot completely guarantee residents from the virus intrusion. At present, the injection of COVID-19 vaccine is the most direct and effective preventive way to prevent viral infections [3]. Currently, governments, medical institutions and scientific research companies of various countries have worked tirelessly to develop vaccines to control the spread of COVID-19 [4]. 

In fact, as claimed by WHO, before the COVID-19 vaccine is launched on a large scale, among the protective measures, wearing masks, especially N95 masks, is an effective way to reduce COVID-19 spread and infection rate. It is estimated that wearing masks can reduce the spread of COVID-19 by more than 70% [5]. Given the significance of wearing masks, it is important to encourage residents to purchase and wear masks against COVID-19. However, residents need to pay a certain cost to purchase masks, and then a financial burden will be placed on them. As a result, before encouraging residents to purchase and wear masks, it is necessary to understand whether residents are willing to pay for purchasing masks against COVID-19, the value of willingness to pay (WTP) and the related determinants. Because COVID-19 is an emergency in public health, limited research attention has been focused on residents’ WTP for purchasing mask against COVID-19. To close the research gap and provide recommendations to government agencies, this research aims to use survey data collected in China to analyze residents’ WTP for purchasing mask against COVID-19 by using contingent valuation method.

In the public health research domain, protection motivation theory is a commonly used theory to understand individual’s self-protective behavior and protective behavior decision making, and its effectiveness has been validated [6]. In this research, purchasing masks against COVID-19 is a self-protective behavior and whether and how much willing to pay for purchasing mask against COVID-19 is protective behavior decision making. Hence, protection motivation theory can be regarded as the theoretical basis in the current research, and WTP can be incorporated into the protection motivation theory conceptual framework. Meanwhile, as a new virus, residents’ knowledge about COVID-19 can have an effect on residents’ risk perception and subsequent behavioral responses. Lack of knowledge about COVID-19 may provide space for rumors to breed and spread, and induce residents to take incorrect protective measures. Hence, residents’ knowledge about COVID-19 has been considered and added into the research framework. The main contribution of this research is that we systematically explored whether residents are willing to pay for purchasing mask against COVID-19, the value of WTP and the related determinants. To the best of our knowledge, this is the first attempts to understand residents’ WTP for purchasing mask against COVID-19, which can further enrich the research on COVID-19 and provide implications for government agencies to make intervention strategies. 

## 2. Literature Review 

### 2.1. Protection Motivation Theory

In behavioral research context, several theoretical models and theories are well developed to explore individual’s behavioral reaction, such as theory of planned behavior, cognitive dissonance theory, value-belief-norm theory, norm activation model, protection motivation theory, etc. Among them, protection motivation theory (PMT), which was originally developed by [6], has been frequently adopted to explore why individuals take protective actions. PMT has been widely used in various crisis and health risk situations (e.g., natural disasters, climate change, epidemics) and its explanatory power has been validated [6,7,8,9].

According to PMT, the aim of an individual to take protective actions is to avoid or reduce the negative influences of potential risks [6,7]. The decision to take protective actions is influenced by two factors: threat appraisal and coping appraisal [7]. Threat appraisal refers to individuals tending to assess the negative outcomes of the potential risks and the threats on themselves such as whether they are easily affected by potential risks and whether the negative effects of potential risks are severe [6]. In general, threat appraisal can be divided into two sub-variables: perceived severity and perceived vulnerability. The more severity and vulnerability individuals perceived, the more likely they are willing to take protective actions to prevent risks. Coping appraisal refers to individuals tending to assess whether they have ability and capability to take protective actions and whether the protective actions can help them avoid or lower the adverse outcome of risks [6]. Coping appraisal can be divided into two sub-variables: self-efficacy and response efficacy. PMT assumes that the higher the self-efficacy and response efficacy, the more likely individuals are prepared to take protective actions to prevent risks.

In the COVID-19 research domain, research has been done to explore residents’ risk perceptions of COVID-19 and protective behaviors. Based on PMT, Ref. [10] assessed public risk perception of COVID-19 and its impact on taking protective actions, and further examined the predictors of risk perception. They found that the level of public risk perception is relatively high, and risk perception is positively correlated with the adoption of protective actions. Personal experience with the virus, trust in government, prosocial value, personal knowledge and personal efficacy are all significant predictors of risk perception. Ref. [11] investigated the perception of risk and the worries about COVID-19 infection in both healthcare workers and the general population in Italy. They found that healthcare workers report higher risk perception and more eager to take protective measures. Ref. [12] used PMT to explore the immediate risk perceptions and psychological effects of the COVID-19 pandemic among Italian participants. They noted that perceived control, perceived efficacy of containment measures and affective states affect risk perception, which further promote residents to take protective behaviors. Ref. [13] adopted PMT to analyze the motivations of residents to take protective measures and behaviors during the early phase of COVID-19. They found that risk perception can drive the adoption of protective behaviors. Ref. [14] investigated the effect of risk perception on self-reported engagement in protective behaviors based on the research samples from United States. They noted that the samples demonstrate growing awareness of risk and report engaging in protective behaviors with increasing frequency. Their research findings highlighted the importance of risk perception and further confirmed the positive relationship between risk perception and protective behavior. Ref. [15] examined the effect of the components of PMT on taking protective health behaviors related to the COVID-19 virus. They found that the components of PMT are positively and significantly associated with the adoption of protective health behaviors. Other studies such as [16,17,18] also explored residents’ risk perceptions of COVID-19 and protective behaviors, and proposed measures to encourage residents to take protective behaviors to impede the spread of COVID-19. 

Though prior research has explored the issues about residents’ risk perceptions of COVID-19 and protective behaviors, limited research has been performed to explore residents’ WTP for taking protection actions such as purchasing mask against COVID-19 and examined the related determinants. Thus, to enrich the research on COVID-19, the current research aims to explore the determinants and WTP for purchasing masks against COVID-19. Meanwhile, given the popularity and explanatory power, PMT has been selected as the theoretical basis of this research.

### 2.2. Willingness to Pay and Contingent Valuation Method

In essence, the aim of residents to purchase and wear masks against COVID-19 is to avoid infection by COVID-19 and keep the virus at bay. Health is a non-market product and cannot be traded in the market. Hence, its value cannot be decided by demand and supply functions. According to microeconomic theory, the value formation of non-market products is largely decided by how much residents are willing to pay. In this research, residents’ WTP refers to how much they intend to spend on purchasing masks against COVID-19. 

In practice, there are two methods to obtain information about WTP and calculate the WTP value: choice experiment method and contingent valuation method. Compared with choice experiment method, contingent valuation method is more robust and easy to understand and implement. Contingent valuation method is a typical stated preference evaluation method based on the utility maximization theory to assess the economic value of non-market products by asking the respondents’ WTP directly [19,20]. The respondents’ answer is based on their own ideas rather than on their previous purchase behavior. The implicit assumption of this method is that the respondents can clearly understand their preferences and express them truthfully during the inquiry process [21]. Currently, contingent valuation method is the most widely used and effective method to analyze WTP and calculate the WTP value [21]. For this consideration, contingent valuation method has been adopted to analyze residents’ WTP for purchasing mask against COVID-19. 

For contingent valuation method, there are four elicitation techniques to inquire the respondents’ WTP information: bidding game, open-ended, payment card and dichotomous choice [22]. Bidding game elicitation technique refers to setting an initial bid value first and then continuously increase or decrease the price level to detect respondents’ WTP [23]. This elicitation technique is greatly affected by the initial bid value, and the entire game process requires constant communication with the respondents, which consumes a lot of time and effort. Hence, in practice, bidding game elicitation technique is rarely adopted. Open-ended elicitation technique refers to let respondents state their WTP directly [24]. Though this elicitation technique is simple and easy to conduct, it will also cause respondents to not know how to answer or even not to answer, which in turn affects the reliability of the results [24]. Payment card elicitation technique refers to setting different WTP interval first and then ask respondents to state their WTP based on these intervals [25]. Compared with open-ended elicitation technique, this elicitation technique is convenient for respondents to state their WTP. It should be cautioned that the credibility of the results depends largely on the rationality of the interval settings. Dichotomous choice elicitation technique refers to setting an initial bid value first and then inquire respondents whether they are willing to pay the initial bid value [26]. This elicitation technique simulates the real market behavior, but it requires a very complex probability statistical model to estimate the WTP value. 

## 3. Data and Method 

### 3.1. Questionnaire Design 

Given that N95 masks are more effective to curb COVID-19 spread than cloth masks and surgical masks, the survey aims to explore residents’ WTP for buying N95 masks. The masks mentioned in the following are all refer to N95 masks. Questionnaire survey was performed to collect residents’ WTP information. The questionnaire included 4 parts. The first part briefly introduced the research background and research purpose, and ensured security of the primary data [27]. The second part aimed to obtain the information related to respondents’ knowledge about COVID-19 and the four variables of PMT.

Multiple measurement items were employed to measure 5 variables mentioned above. All the items were assessed on a five-point Likert scale ranging from 1 (completely disagree) to 5 (completely agree), and respondents were asked to evaluate the items based on their own feelings and perceptions. To ensure the validity of the measurement items, all of them were adapted from prior research and slightly modified to make them applicable for the current research context. Specifically, the measurement items of the four variables of PMT were adapted from [16,17,19], and each variable was measured using three items. Four items adapted from [10,24,28] were employed to measure knowledge about COVID-19. To ensure the accuracy of these items, 7 academic scholars whose research interests focus on health behavior and risk communication were invited to help us to refine these items. Based on their comments, minor revisions such as typos and phrasing were made [29]. The final items of the research variables were presented in Appendix A. 

The third part of the questionnaire was used to obtain information about residents’ WTP. We first inquired the respondents whether they are willing to pay for purchasing masks against COVID-19. If not, we asked the respondents to choose from five statements the one that most closely resembled their reason for not being willing to pay. If yes, we then asked them how much they are willing to pay for purchasing masks against COVID-19 per month during the epidemic. To facilitate respondents to express their WTP value, payment card elicitation technique was adopted. In total, we set 8 intervals of WTP value: (1) ¥1–30 ($0.15–4.65); (2) ¥31–60 ($4.80–9.29); (3) ¥61–90 ($9.45–13.94); (4) ¥91–120 ($14.10–18.59); (5) ¥121–150 ($18.74–23.24); (6) ¥151–180 ($23.39–27.88); (7) ¥181–210 ($28.04–32.53) and (8) ¥211–240 ($32.68–37.18). The fourth part was used to collect respondents’ demographic information such as age, gender, family size, health status, etc. [13,30,31].

### 3.2. Data Collection 

In general, there are three commonly used methods to perform questionnaire surveys: online survey method, face to face survey method, and telephone and e-mail survey method [32,33,34,35]. In this research, online survey method was adopted to perform the survey. The reasons can be stated as follows. First, the current survey was performed during the COVID-19 outbreak period. Hence, face to face survey method should not be considered. Second, the response rate of telephone and e-mail survey method was low and time-consuming. Thus, telephone and e-mail survey methods were not considered. 

With the help of Questionnaire Star (www.wjx.cn (accessed on 1 January 2020)), a popular and professional online survey website in China, the questionnaire survey was performed during 1–24 February 2020 in Hubei, Anhui, Jiangsu, Zhejiang, Shandong, Guangdong and Fujian Provinces and the Municipality of Shanghai. Participants were recruited from a large sample pool maintained by the Questionnaire Star. We totally received 3471 questionnaires. After abandoning the invalid questionnaires, 3148 valid questionnaires were obtained and used to perform data analysis. 

## 4. Data Analysis and Results 

In the following data analysis, we first conducted the reliability and validity analysis [36]. Then, we introduced the demographic information of respondents and conducted descriptive analysis to describe the research data. At last, we used the relevant estimation method to identify the determinants of WTP and WTP value. Furthermore, Hubei is the province most affected by the COVID-19 in China, respondents’ perception and attitude toward COVID-19 in Hubei Province may be different from respondents in other provinces. Hence, to better and fully understand the determinants and the value of WTP for purchasing mask against COVID-19, the total valid samples (*N* = 3148) have been divided into 2 sub-samples: Hubei Sample (*N* = 1103) and Non-Hubei (e.g., Anhui, Jiangsu, Zhejiang, Shandong, Guangdong and Fujian Provinces and the Municipality of Shanghai) Sample (*N* = 2045). 

### 4.1. Reliability and Validity Analysis

The results of reliability and validity analysis were presented in Table 1. According to Table 1, it can be concluded that the values of Cronbach’s alpha and composite reliability (CR) were all greater than the threshold value of 0.70 [37]. Thus, the reliability of the survey was acceptable. Furthermore, the factor loadings of the items were all larger than 0.70 and significant (*p* < 0.001), and AVE values also exceeded 0.50 (See Table 1). Thus, the convergent validity of the survey was satisfactory [37]. In addition, as shown in Table 2, the square root of AVE for each variable was greater than its correlation coefficients with the other variables, revealing good discriminant validity [37]. Meanwhile, the Heterotrait–Monotrait (HTMT) Ratio for each of the variables was smaller than 0.85, which further suggested that the discriminant validity of the survey was acceptable [38]. 

### 4.2. Demographic Information

The demographic information of the respondents was illustrated in Table 3. In the total sample, compared with males, females accounted for the largest proportion, suggesting that females are more concerned about COVID-19 and willing to engage in the survey. The respondents were young and middle-aged. More than half of the respondents were aged between 26 and 50. Nearly 80% of the respondents had more than 10 years of education. Most respondents were middle class. Nearly 70% of the respondents earned a monthly income between ¥5000–15,000 ($774–2322). More than 80% of the respondents had 2–5 family members. Nearly half of the respondents acknowledged that their health status is acceptable. Table 3 also presented the demographic information of the respondents in Hubei Sample and Non-Hubei Sample. 

### 4.3. Descriptive Analysis 

There were five main variables in this research: knowledge about COVID-19, perceived severity, perceived vulnerability, self-efficacy and response efficacy. Table 4 introduced the descriptive analysis results of variables. It indicated that the mean values of these five variables are all larger than the average value of 3. Compared with the mean values of four variables in PMT, the mean value of COVID-19 knowledge is relatively lower. Table 4 also presented the mean values of these five variables in Hubei Sample and Non-Hubei Sample. To compare whether there are differences between the mean values of these five variables in Hubei Sample and Non-Hubei Sample, ANOVA analysis was conducted. The results indicated that there are significant differences between the mean values of knowledge about COVID-19 (T = 2.81, *p* < 0.05), perceived severity (T = 3.01, *p* < 0.05), perceived vulnerability (T = 3.11, *p* < 0.05), self-efficacy (T = 2.89, *p* < 0.05) and response efficacy (T = 4.31, *p* < 0.01) in Hubei Sample and Non-Hubei Sample, and the mean values of these five variables in Hubei Sample were significantly higher than that in Non-Hubei Sample. Indeed, compared with respondents in Non-Hubei Sample, respondents in Hubei Sample will definitely pay more attention to COVID-19. Thus, it is understandable that the respondents in Hubei Sample have a higher level of COVID-19 knowledge, and perceive a higher level of severity, vulnerability, self-efficacy, and response efficacy than respondents in Non-Hubei Sample. 

### 4.4. Determinants of Residents’ WTP

To identify the determinants of residents’ WTP for purchasing masks against COVID-19, we should first analyze whether residents are willing to pay or not. As shown in Table 5, among the 3148 valid samples, 2581 observations are willing to pay for purchasing masks against COVID-19, accounting for 81.99% of the total valid samples. In addition, 567 observations are unwilling to pay for purchasing masks against COVID-19, accounting for 18.01% of the total valid samples. This finding suggested that most residents are willing to pay for purchasing masks against COVID-19. Furthermore, Table 5 also presented residents’ WTP for purchasing masks against COVID-19 in Hubei Sample and Non-Hubei Sample. It can be found that the ratio of willing to pay (90.03%) in Hubei Sample is much higher than that in Non-Hubei Sample (79.95%). 

Moreover, the reasons why residents were unwilling to pay for purchasing masks against COVID-19 were explored as well. We summarized five reasons: (1) I am healthy and have strong immunity, so I am unwilling to spend money to purchase and wear masks; (2) I always stay at home, so I am unwilling to spend money to purchase and wear masks; (3) COVID-19 will end soon, so I am unwilling to spend money to purchase and wear masks; (4) I have no money to purchase masks and (5) the cost of purchasing masks should be paid by the government. The frequency and ratio of each reason was shown in Table 6. Table 6 suggested that the reasons why respondents are unwilling to pay for purchasing masks against COVID-19 mainly focus on two aspects: good health and strong immunity, and always stay at home. This finding was also applicable for respondents in Hubei Sample and Non-Hubei Sample. 

To identify the determinants of residents’ WTP, we should select the appropriate estimation model and method. The dependent variable, namely residents’ WTP, was a discrete variable or “0–1” variable. Traditional linear regression estimation method such as ordinary least squares (OLS) estimation method cannot accurately predict the effects of independent variables on dependent variable [39]. Following the suggestion of [40], binary logistic regression method was used. Furthermore, given that residents’ WTP varies with the respondents’ demographic factors such as age, gender, education, income, family size and health status, we introduced these factors as the control variables into the regression analysis. Specifically, *gender* is a dummy variable, which is equal to 1 if the respondent is male and 0 if the respondent is female. *Age* is a continuous variable, which refers to the actual age of the respondent. *Education* is a continuous variable, which refers to the number of years the respondent has received education in school. *Income* is a continuous variable, which refers to the respondent’s household monthly income. *Family size* is a continuous variable, which refers to the number of family members of the respondent. Health status is a dummy variable, which is equal to 1 if the health status is acceptable and very well, and 0 if the health status is not well. 

The results of logistic regression analysis were presented in Table 7. Model 1 showed the regression results of the total sample. For control variables, gender is negatively and significantly associated with residents’ WTP. Family size is positively and significantly associated with residents’ WTP. Other control variables such as age, education, income and health status have no significant effect on residents’ WTP. Residents’ knowledge about COVID-19 (β = 0.345, *p* < 0.05), perceived severity (β = 0.465, *p* < 0.001), perceived vulnerability (β = 0.321, *p* < 0.05), self-efficacy (β = 0.238, *p* < 0.01) and response efficacy (β = 0.317, *p* < 0.05) are all positively and significantly associated with residents’ WTP. Among the standardized coefficients, the standardized coefficient of perceived severity is the largest. To further evaluate the proportional contribution of each variable to the explained variance, we used the method proposed by [41,42] to perform a relative importance analysis. Table 8 presented the results of the relative weight of the importance analysis of the determinants of residents’ WTP. As shown in Table 8, perceived severity explains a larger percentage of the variance (20.49%) towards residents’ WTP than any other variables. Thus, by comparing the relative contribution of each variable towards the predictive criterion of residents’ WTP, it can be concluded that perceived severity has the largest effect on residents’ WTP. 

Models 2 and 3 in Table 7 showed the regression results of Non-Hubei Sample and Hubei Sample. Overall, the findings in Non-Hubei Sample (Model 2) and Hubei Sample (Model 3) were in line with the findings in total sample (Model 1). Compared with Non-Hubei Sample (Model 2), the standardized coefficients of knowledge about COVID-19, perceived severity, perceived vulnerability, self-efficacy and response efficacy in Hubei Sample (Model 3) are relatively larger. Meanwhile, it is worth noting that all the control variables in Hubei Sample (Model 3) are insignificant. 

### 4.5. Determinants of Residents’ WTP Value

To identify the determinants of residents’ WTP value, we should analyze the distribution of residents’ WTP value. The distribution of residents’ WTP value was presented in Table 9. Prior research such as [43,44,45] noted that WTP value can be decided based on the median value of each interval. Hence, the median value of each interval can also be regarded as a reasonable representation level of residents’ WTP value in this research. On the basis of residents’ WTP value, we calculated the average value of residents’ WTP value. By referring to the work of [45,46], the average value of residents’ WTP value can be expressed as follows:

(1) $$\mathrm{WTP}=\sum_{i=1}^{n}{\mathrm{WTP}}_{i}\frac{N_{i}}{N}$$

In Equation (1), WTP means average value of residents’ WTP value, ${\mathrm{WTP}}_{i}$ means the WTP value of respondents who select *i*th interval, $N_{i}$ means the number of respondents who select *i*th interval and *N* means the total number of respondents who are willing to pay. According to Equation (1) and Table 9, the average value of residents’ WTP value for purchasing masks against COVID-19 is ¥120.92 ($18.73) per month during the epidemic in Hubei province and it is ¥100.16 ($15.50) per month during the epidemic in non-Hubei province. Overall, the average value of residents’ WTP value for purchasing masks against COVID-19 is ¥106.53 ($16.51) per month during the epidemic nationally. 

In this research, the dependent variable, namely residents’ WTP value, was measured by a payment card elicitation technique. Residents’ WTP value was an interval value. To improve the prediction accuracy of the estimation model, the interval regression estimation model was employed [39,47]. The estimation results were presented in Table 10. 

As shown in Table 10, in the total sample (Model 4), residents’ COVID-19 knowledge (β = 0.221, *p* < 0.05), perceived severity (β = 0.263, *p* < 0.05), perceived vulnerability (β = 0.280, *p* < 0.01) and response efficacy (β = 0.401, *p* < 0.001) are positively and significantly associated with residents’ WTP value, while self-efficacy has no significant effect on residents’ WTP value (β = 0.347, *p* > 0.05). As for control variables, gender (β = −0.181, *p* < 0.01) and health status (β = −0.237, *p* < 0.05) are negatively and significantly associated with residents’ WTP value. Other control variables such as age, education, income and family size have no significant effect on residents’ WTP value. In Non-Hubei Sample (Model 5) and Hubei Sample (Model 6), the research results are almost consistent with findings in total sample (Model 4). However, in Hubei Sample (Model 6), all control variables have no significant effect on residents’ WTP value. 

## 5. Discussion

We undertook the current research with the purpose to explore the determinants of residents’ willingness to pay (WTP) for purchasing masks against COVID-19 and the value of WTP. Based on protection motivation theory, we demonstrated that perceived severity, perceived vulnerability, self-efficacy and response efficacy are the positive antecedents of residents’ WTP. These findings revealed that when residents perceive a higher level of severity and vulnerability, and have a higher level of self-efficacy and response efficacy, they will be likely to pay for purchasing masks against COVID-19. The current research findings were consistent with prior research on COVID-19 such as [10,12,15], which further highlighted the importance of protection motivation theory in explaining residents’ protective behaviors during the COVID-19 pandemic. Meanwhile, this research also identified the positive impact of residents’ knowledge about COVID-19 on residents’ WTP, indicating that the more residents are knowledgeable about COVID-19, the more likely they are to pay for purchasing masks against COVID-19. This finding mirrored prior research findings [48] and further accented the significance of public COVID-19 knowledge in COVID-19 research. 

By comparing the standardized coefficients of these five variables, it can be found that the standardized coefficient of perceived severity is the largest, revealing that perceived severity has the largest effect on residents’ WTP. Indeed, the primary reason why residents are willing to pay for purchasing masks against COVID-19 is that they perceive the severity of COVID-19 and wish to purchase and wear masks to reduce the negative impact of COVID-19 on them. For control variables, gender negatively impacts residents’ WTP, suggesting that females are more willing to pay for purchasing masks against COVID-19 than males. While family size positively impacts residents’ WTP, uncovering that the more family members, the more likely they are to pay for purchasing masks against COVID-19. Other control variables such as age, education, income and health status have no significant effect on residents’ WTP. This may be due to the fact that COVID-19 has spread rapidly throughout the world and its negative outcome on human society has been known to the public [49]. Thus, anyone, regardless of age, education, income and health status, is all willing to pay for purchasing masks against COVID-19.

Furthermore, the determinants of the value of WTP for purchasing masks against COVID-19 were also explored. Findings showed that residents’ COVID-19 knowledge, perceived severity, perceived vulnerability and response efficacy are the positive antecedents of WTP value. These findings indicated that when residents are knowledgeable about COVID-19, perceive a higher level of severity and vulnerability, and have a higher level of response efficacy, they are likely to pay more for purchasing masks against COVID-19. However, contrary to our expectation, this research indicated that self-efficacy has no significant effect on WTP value. This may be because that the cost paid on purchasing masks against COVID-19 is not too much and nearly all residents can afford it. That is, residents have ability and capacity to purchase masks against COVID-19 and thus the effect of self-efficacy is insignificant. Given that prior COVID-19 literature rarely discussed residents’ WTP for taking protective behaviors especially purchasing and wearing mask, this research contributed to existing body of knowledge on COVID-19 and residents’ protective behaviors literature, thus advancing the science in this research domain for other scholars to explore. 

As for control variables, gender and health status negatively affect residents’ WTP value, suggesting that female and residents in poor health are more likely to pay more for purchasing masks against COVID-19. Other control variables such as age, education, income and family size have no significant effect on residents’ WTP value, revealing that anyone, regardless of age, education, income and family size, is willing to pay for purchasing masks against COVID-19. 

## 6. Conclusions and Limitations 

COVID-19 has shown a pandemic trend globally. To curb its spread and reduce its negative effect on the global economy, especially on public health, all circles in the world should take measures to against COVID-19 [49]. From an individual perspective, this research focused on residents’ self-protective behaviors and aimed to examine the determinants and willingness to pay for purchasing mask against COVID-19. 

This research found that most residents are willing to pay for purchasing masks against COVID-19. Residents’ COVID-19 knowledge, perceived severity, perceived vulnerability, self-efficacy and response efficacy are positively and significantly associated with residents’ WTP, and perceived severity has the largest effect. For control variables, only gender and family size are significantly associated with residents’ WTP. Other control variables such as age, income, education and health status have no significant effect on residents’ WTP. Furthermore, we also examined the determinants of residents’ WTP value. The results indicated that residents’ COVID-19 knowledge, perceived severity, perceived vulnerability and response efficacy are positively and significantly associated with residents’ WTP value. However, self-efficacy has no significant effect on residents’ WTP value. Gender and health status are significantly associated with residents’ WTP value. Other control variables such as age, education, income and family size have no significant effect on residents’ WTP value. 

In addition, we also calculated the average value of residents’ WTP value for purchasing mask against COVID-19. The average value of residents’ WTP value for purchasing mask against COVID-19 is ¥106.53 ($16.51) per month during the epidemic nationally. Meanwhile, this research further noted that residents in Hubei province tending to pay more than residents in non-Hubei province. The average value of residents’ WTP value for purchasing mask against COVID-19 is ¥120.92 ($18.73) per month during the epidemic in Hubei province, and it is ¥100.16 ($15.50) per month during the epidemic in non-Hubei province. 

There are several limitations in this research. First, an online survey method was adopted to collect research data. In general, face to face survey method is more applicable for using contingent value method. In the following research, face to face survey methods can be used. Second, payment card elicitation technique was used in this research. The WTP value largely depends on the interval value we set. Hence, it needs caution to generalize the residents’ WTP value. Third, the research sample of this study was still limited, which may affect the research findings. In future research, more data can be gathered to replicate the current research findings. Finally, we did not consider the data from 2021. In future research, the data from 2021 should be collected and analyzed.

## Figures and Tables

**Table 1:** Reliability and validity analysis.

Variables	Item	Loading	Cronbach’s Alpha	CR	AVE
Knowledge	KN1	0.85 ***	0.80	0.89	0.68
	KN2	0.77 ***			
	KN3	0.84 ***			
	KN4	0.83 ***			
Perceived severity	PS1	0.88 ***	0.82	0.88	0.71
	PS2	0.83 ***			
	PS3	0.81 ***			
Perceived vulnerability	PV1	0.79 ***	0.77	0.84	0.64
	PV2	0.82 ***			
	PV3	0.79 ***			
Self-efficacy	SE1	0.79 ***	0.83	0.89	0.72
	SE2	0.89 ***			
	SE3	0.87 ***			
Response efficacy	RE1	0.81 ***	0.79	0.86	0.67
	RE2	0.79 ***			
	RE3	0.86 ***			

**Note:** *** indicates significant at 0.1% significance level.

**Table 2:** Discriminant validity analysis.

Variables	KN	PS	PV	SE	RE
KN	**0.82**	0.42	0.63	0.57	0.49
PS	0.53	**0.84**	0.37	0.53	0.38
PV	0.48	0.35	**0.80**	0.49	0.51
SE	0.55	0.43	0.43	**0.85**	0.50
RE	0.41	0.45	0.38	0.33	**0.82**

**Note:** The bold values (diagonal elements) are the square root of AVE values; the values below the diagonal are the correlation coefficients among variables; the values above the diagonal are the Heterotrait–Monotrait (HTMT) Ratio of each variable.

**Table 3:** Samples’ demographic information.

Category		Total Sample		Non-Hubei Sample		Hubei Sample	
		*N*	%	*N*	%	*N*	%
Gender	Female	1701	54.03%	1093	53.45%	582	52.77%
	Male	1447	45.97%	952	46.55%	521	47.23%
Age	18–25	564	17.92%	350	17.11%	190	17.23%
	26–40	723	22.97%	481	23.52%	250	22.67%
	41–50	941	29.89%	623	30.46%	330	29.92%
	51–60	503	15.98%	318	15.55%	173	15.68%
	>60	417	13.25%	273	13.35%	160	14.51%
Years of education	≤6	314	9.97%	218	10.66%	121	10.97%
	7–9	316	10.04%	181	8.85%	120	10.88%
	10–12	789	25.06%	519	25.38%	271	24.57%
	13–16	1045	33.20%	691	33.79%	369	33.45%
	≥17	684	21.73%	436	21.32%	222	20.13%
Monthly Income	<¥5000 ($774)	409	12.99%	251	12.27%	162	14.69%
	¥5000–10,000 ($1548)	1101	34.97%	731	35.75%	381	34.54%
	¥10,001–15,000 ($2322)	1070	33.99%	701	34.28%	362	32.82%
	>¥15,000	568	18.04%	362	17.70%	198	17.95%
Family size	1	220	6.99%	139	6.80%	75	6.80%
	2–3	1448	46.00%	921	45.04%	516	46.78%
	4–5	1196	37.99%	775	37.90%	408	36.99%
	>5	284	9.02%	210	10.27%	104	9.43%
Health status	Not well	1045	33.20%	690	33.74%	370	33.54%
	Acceptable	1479	46.98%	952	46.55%	501	45.42%
	Very well	624	19.82%	403	19.71%	232	21.03%
Observations		3148		2045		1103	

**Table 4:** Descriptive analysis result of research variable.

Variables	Total Sample		Non-Hubei Sample		Hubei Sample	
	Mean	SD	Mean	SD	Mean	SD
Knowledge (KN)	3.639	0.518	3.501	0.683	3.973	0.732
Perceived severity (PS)	4.219	0.457	4.093	0.683	4.472	0.702
Perceived vulnerability (PV)	4.313	0.537	4.101	0.781	4.598	0.692
Self-efficacy (SE)	4.411	0.531	4.278	0.602	4.693	0.573
Response efficacy (RE)	4.298	0.633	4.128	0.821	4.601	0.721

**Note:** SD = Standard Deviation.

**Table 5:** Residents’ WTP.

Response	Total Sample		Non-Hubei Sample		Hubei Sample	
	Frequency	Ratio	Frequency	Ratio	Frequency	Ratio
Willing to pay	2581	81.99%	1635	79.95%	993	90.03%
Unwilling to pay	567	18.01%	410	20.05%	110	9.97%
Observations	3148		2045		1103	

**Table 6:** Reasons of unwilling to pay.

Reason	Total Sample		Non-Hubei Sample		Hubei Sample	
	Frequency	Ratio	Frequency	Ratio	Frequency	Ratio
I am healthy and have strong immunity	210	37.04%	140	34.15%	52	47.27%
I always stay at home	165	29.10%	120	29.27%	31	28.18%
COVID-19 will end soon	80	14.11%	50	12.20%	20	18.18%
I have no money	32	5.64%	25	6.10%	1	0.91%
The cost should be paid by the government	80	14.11%	75	18.29%	6	5.45%
Observations	567		410		110	

**Table 7:** Determinants of residents’ WTP.

Variables	Logistic Regression		
	Total Sample	Non-Hubei Sample	Hubei Sample
	Model 1	Model 2	Model 3
Knowledge	0.345 *	0.281 **	0.389 **
Perceived severity	0.465 ***	0.412 *	0.507 ***
Perceived vulnerability	0.321 *	0.318 **	0.397 *
Self-efficacy	0.238 **	0.221 *	0.313 *
Response efficacy	0.317 *	0.291 ***	0.403 ***
Gender	−0.131 **	−0.187 **	0.149
Age	0.131	0.123	0.199
Education	0.204	0.253	0.101
Income	0.181	0.153	0.271
Family size	0.208 *	0.196 **	0.314
Health status	0.217	0.253	0.318
Observations	3148	2045	1103
LR χ^2^	130.218	123.679	141.783
Prob > χ^2^	0.000	0.000	0.001
Pseudo R^2^	0.554	0.511	0.589
Log Likelihood	−269.341	−291.327	−245.827

**Notes:** * <0.05, ** <0.01 and *** <0.001.

**Table 8:** Relative importance weights of the determinants of residents’ WTP.

Variables	Raw Relative Weight	Rescaled Relative Weight
Knowledge	0.091	16.49%
Perceived severity	0.113	20.49%
Perceived vulnerability	0.081	14.77%
Self-efficacy	0.061	11.02%
Response efficacy	0.054	9.56%
Gender	0.018	3.18%
Age	0.026	4.74%
Education	0.028	5.11%
Income	0.024	4.18%
Family size	0.025	4.46%
Health status	0.033	6.01%
Pseudo R^2^	0.554	100%

**Table 9:** Distribution of residents’ WTP value.

Interval	Total Sample		Non-Hubei Sample		Hubei Sample	
	Frequency	Ratio	Frequency	Ratio	Frequency	Ratio
¥1–30	288	11.16%	225	13.76%	70	7.05%
¥31–60	422	16.35%	345	21.10%	70	7.05%
¥61–90	255	9.88%	195	11.93%	55	5.54%
¥91–120	481	18.64%	245	14.98%	260	26.18%
¥121–150	592	22.94%	299	18.29%	300	30.21%
¥151–180	241	9.34%	91	5.57%	130	13.09%
¥181–210	187	7.25%	125	7.65%	60	6.04%
¥211–240	115	4.46%	110	6.73%	48	4.83%
Observations	2581		1635		993	

**Table 10:** Determinants of residents’ WTP value.

Variables	Total Sample	Non-Hubei Sample	Hubei Sample
	Model 4	Model 5	Model 6
Knowledge	0.221 *	0.309 ***	0.268 **
Perceived severity	0.263 *	0.197 **	0.331 ***
Perceived vulnerability	0.280 **	0.251 **	0.342 *
Self-efficacy	0.347	0.202	0.307
Response efficacy	0.401 ***	0.344 **	0.413 ***
Gender	−0.181 **	−0.197 *	−0.287
Age	−0.207	−0.183	−0.257
Education	0.231	0.253	0.186
Income	0.136	0.187	0.256
Family size	−0.260	−0.183	0.259
Health status	−0.237 *	−0.209 **	−0.289
Observations	2581	1635	993
Wald χ^2^	97.359	92.257	89.183
Prob > χ^2^	0.000	0.000	0.000
Log Likelihood	−1123.387	−1242.207	−1037.342

**Notes:** * <0.05, ** <0.01 and *** <0.001.

## Data Availability

The datasets used and/or analyzed during the current study are available from the corresponding author on reasonable request.

## Appendix A. Variable and Measurement Item

**Table:**

Variables	Measurement Item
Knowledge (KN)	KN1: COVID-19 is a respiratory infection caused by a new species of coronavirus family
	KN2: COVID-19 can be transmitted through respiratory droplets such as cough and sneeze
	KN3: COVID-19 can be prevented through wearing mask and personal hygiene
	KN4: The common symptoms of COVID-19 are fever, cough and shortness of breath
Perceived severity (PS)	PS1: COVID-19 is a serious social issue
	PS2: COVID-19 will have negative consequences
	PS3: The negative effect of COVID-19 is severe
Perceived vulnerability (PV)	PV1: COVID-19 can negatively impact me
	PV2: I am vulnerable to the negative effects of COVID-19
	PV3: My chances of being infected by COVID-19 is high
Self-efficacy (SE)	SE1: It is easy for me to purchase and wear mask
	SE2: If I wanted to, I could easily purchase and wear mask
	SE3: It is mostly up to me whether I purchase and wear mask
Response efficacy (RE)	RE1: Wearing mask can impede the spread of COVID-19
	RE2: Wearing mask can lower the chances of being infected by COVID-19
	RE3: Wearing mask can defeat COVID-19 as soon as possible
